# Personalised neoantigen‐based therapy in colorectal cancer

**DOI:** 10.1002/ctm2.1461

**Published:** 2023-11-03

**Authors:** Ya‐Juan Zhu, Xiong Li, Ting‐Ting Chen, Jia‐Xiang Wang, Yi‐Xin Zhou, Xiao‐Li Mu, Yang Du, Jia‐Ling Wang, Jie Tang, Ji‐Yan Liu

**Affiliations:** ^1^ Department of Biotherapy and Cancer Center State Key Laboratory of Biotherapy West China Hospital Sichuan University Chengdu China; ^2^ Department of Gastroenterology The Second Affiliated Hospital of Xi'an Jiaotong University Xi'an China; ^3^ The Second Clinical Medical College of Lanzhou University Lanzhou China; ^4^ Department of Renal Cancer and Melanoma Peking University Cancer Hospital & Institute Beijing China; ^5^ Clinical Trial Center West China Hospital Sichuan University Chengdu China

**Keywords:** cancer vaccine, colorectal cancer, immunotherapy, neoantigens, targeted therapy

## Abstract

Colorectal cancer (CRC) has become one of the most common tumours with high morbidity, mortality and distinctive evolution mechanism. The neoantigens arising from the somatic mutations have become considerable treatment targets in the management of CRC. As cancer‐specific aberrant peptides, neoantigens can trigger the robust host immune response and exert anti‐tumour effects while minimising the emergence of adverse events commonly associated with alternative therapeutic regimens. In this review, we summarised the mechanism, generation, identification and prognostic significance of neoantigens, as well as therapeutic strategies challenges of neoantigen‐based therapy in CRC. The evidence suggests that the establishment of personalised neoantigen‐based therapy holds great promise as an effective treatment approach for patients with CRC.

## Graphical abstract

Critical Points:

1. Peptide vaccine and neoantigens pulsed DC can trigger a robust antitumor immune response with less emergence of adverse events than other therapeutic regimens.
2. DNA vaccine can spontaneously induce T cell response against tumor and inhibit tumor growth with low cost.
3. mRNA vaccines have been shown to safely induce neoantigen‐specific T‐cell responses without MHC haplotype restriction.
4. Personalized immunotherapy based on neoantigens will be an effective treatments for patients with CRC.

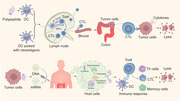

## 1 COLORECTAL CANCER AND NEOANTIGENS

### 1.1 The genetic mutations and molecular subtypes of colorectal cancer

Colorectal cancer (CRC) is a malignant tumour of the digestive system with insidious onset and poor prognosis. It is the third most common cancer in the world.^1^ Despite that significant progress has been made in the early diagnosis and treatment of CRC, and CRC is still a health concern because of the second‐highest mortality rate and huge economic burdens. The development of CRC is characterised by a multistep and asymptomatic progression. It usually originates from benign polyps and is driven by the accumulation of genetic mutations and epigenetic changes, resulting in histological and morphological alternations that eventually evolve into CRC.^2^

The development of CRC mainly involves three signalling pathways, including chromosomal instability (CIN), microsatellite instability (MSI) and CpG island methylation phenotype (CIMP).^3^, ^4^, ^5^ CIN is the most classical and common molecular pathway involved in CRC, accounting for 75% of all CRC cases. It is characterised by chromosomal alterations, including somatic copy number alterations due to deletion, aneuploid loss, insertion and amplification. CIN is often associated with mutations in specific tumour‐suppressor genes and oncogenes (APC, KRAS, PIK3CA, TGF‐β and TP53), which activate crucial pathways involved in the initiation and progression of CRC. MSI is characterised by a hypermutated phenotype caused by deficiencies in the DNA mismatch repair (MMR) system that may occur occasionally due to epigenetic alterations in the MLH1 gene or in the context of Lynch syndrome. In Lynch syndrome, a mutation causes the inactivation of one of the four MMR genes (MLH1, MSH2, MSH6 and PMS2).^5^ The CIMP pathway accounts for 20% of CRC cases. The CIMP has massive hypermethylation of oncogene promoters (CpG island sites). The CIMP can lead to the silencing of tumour‐suppressor genes and loss of protein expression or overlap with the CIN pathway through TP53 mutation and Wnt or TGFβ signalling pathway activations.^1^, ^6^, ^7^

There are a variety of genetic mutations, such as KRAS/NRAS, and BRAF in CRC. Particularly, there is a genomic characteristic leading to the accumulation of mutations, named MSI. The cancer heterogeneity has a significant impact on the diagnosis and treatment of CRC. Therefore, according to the molecular and genetic characteristics of CRC, the consensus molecular subtype CMS was used to classify CRC patients into four different subtypes: CMS1 (MSI Immune), CMS2 (Canonical), CMS3 (Metabolic) and CMS4 (Mesenchymal). This classification method can be used to predict the response of patients to treatment regimens and select the most appropriate treatment for each patient to achieve personalised treatment.^8^, ^9^

### 1.2 Small insertion/deletion contributes to the emergence of neoantigens in CRC

Microsatellite stability (MSS) and mismatch repair process are important factors to determine the therapy strategies to combat malignancies.^10^ Approximately 12%–15% of CRC nodes exhibit mismatch repair deficiency (dMMR), a feature thought to be the primary cause of immunogenic phenotype of MSI. The anti‐cancer immunotherapies mainly include immune checkpoint inhibitors (ICIs), cancer vaccines and adoptive T‐cell transfer. Among these, neoantigen‐based immunotherapy has shown prospects without specific requirements for the patient's MSI status or tumour mutation burden (TMB).^11^, ^12^

**FIGURE 1:**
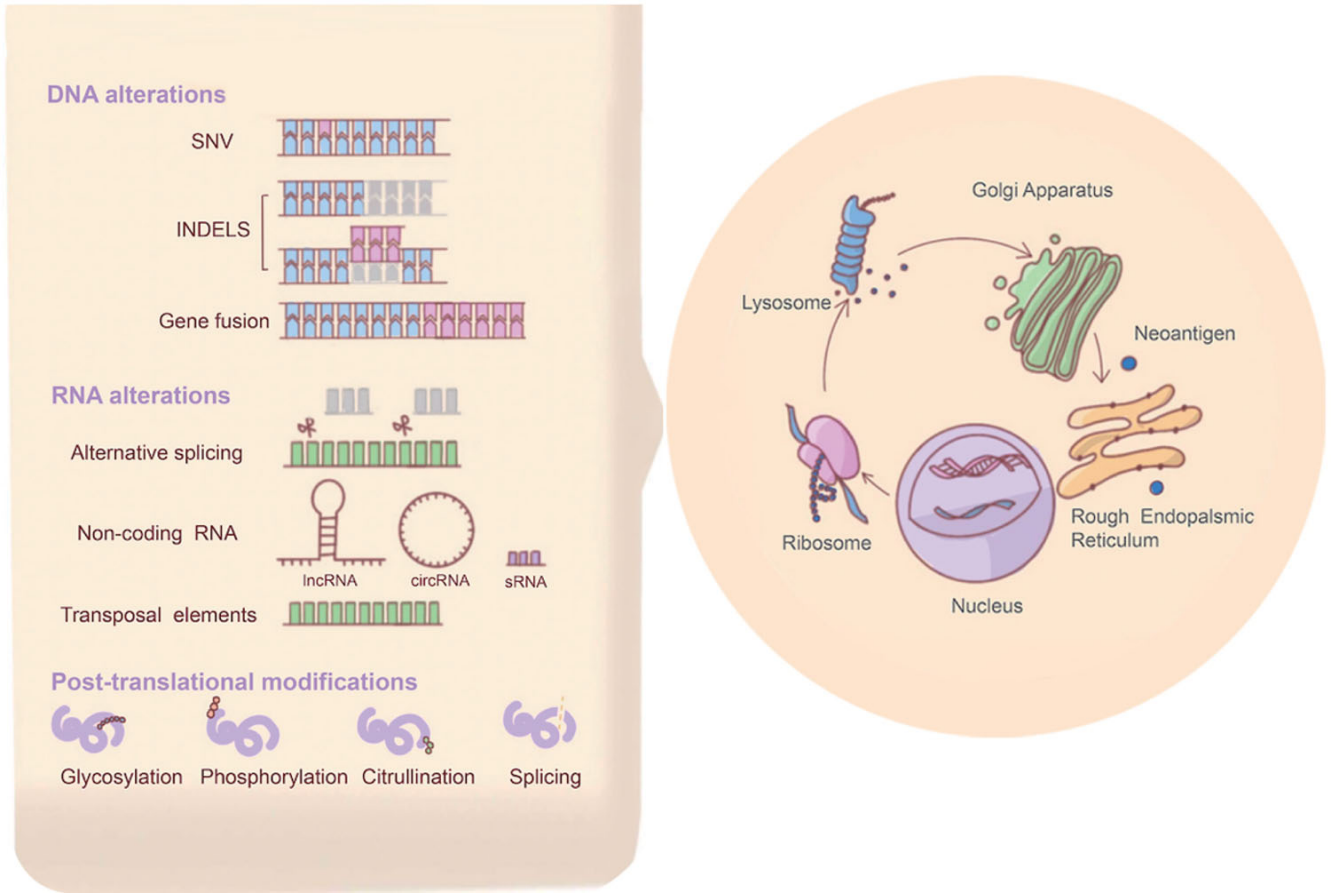
The generation of neoantigens in colorectal cancer (CRC). Neoantigens can be caused by non‐synonymous somatic mutations, such as single‐SNVS, small indels and gene fusion, the posttranscriptional abnormalities and cancer‐specific posttranslational protein modifications, such as methylation, phosphorylation, acetylation and glycosylation. Small insertion/deletion is the most common source of neoantigen production in CRC. SNVS, single‐nucleotide variants; indels, insertions/deletions.

Neoantigens are cancer‐specific abnormal peptides that can be recognised as non‐self, and trigger an immune response in the host. These abnormalities can be caused by non‐synonymous somatic mutations, such as single‐nucleotide variants (SNVS), small insertions/deletions (indels), frameshift mutations or other genomic rearrangements, such as gene fusion. The posttranscriptional abnormalities including cancer‐specific alterations in exon splicing, intron retention and early termination of transcription attribute the occurrence of neoantigens. Neoantigens can also be produced by cancer‐specific posttranslational protein modifications, such as methylation, phosphorylation, acetylation and glycosylation.^13^, ^14^ The most common source of neoantigen production in CRC is small indels (Figure 1). Tumuor antigens can be divided into tumour‐associated antigen (TAA) and tumour‐specific antigens (TSA), also known as neoantigen. TAA is an overexpressed protein in tumour cells, whereas TSA is only expressed by cancer cells. Therefore, the immune system can easily distinguish tumour tissue from healthy tissue, thus minimising the risk of serious adverse events.^15^

**FIGURE 2:**
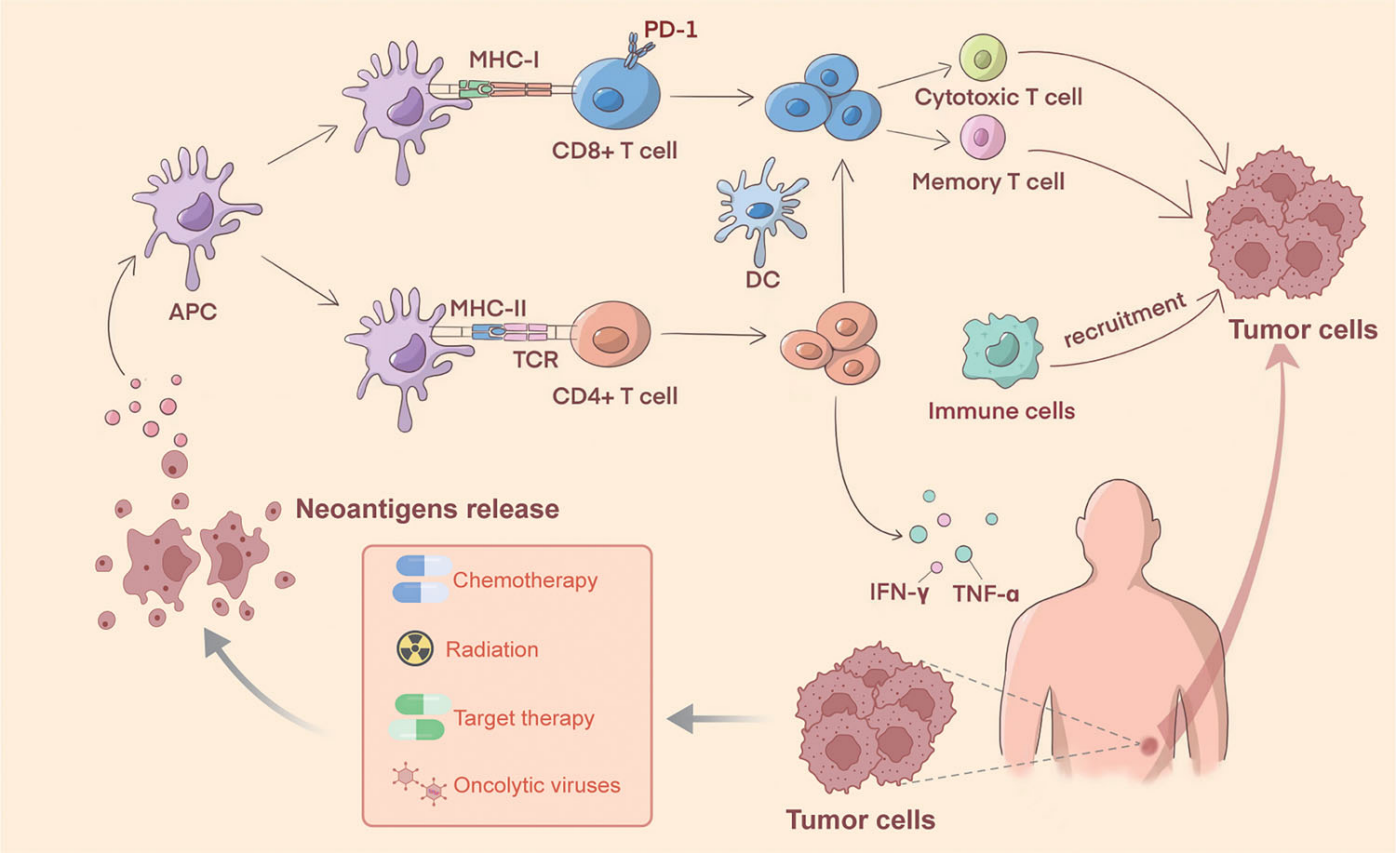
The mechanism of combination therapy and effect of neoantigen‐specific T cells in colorectal cancer (CRC). Traditional therapies, including chemoradiotherapy, antiangiogenic agents and oncolytic viruses, can induce the release of tumour neoantigens and epitope spreading to promote the priming of neoantigen‐specific response. Neoantigens are cancer‐specific abnormal peptides that can be recognised as non‐self, and trigger a host immune response. APC, antigen‐presenting cell; TCR, T‐cell receptor; MHC, major histocompatibility complex; DC, dendritic cell; CTL, cytotoxic lymphocyte; IFN‐γ, interferon‐γ; TNF‐α, tumour necrosis factor‐α.

The Cancer Genome Map illustrates that hypermutant and non‐hypermutant are two sporadic types of CRC. MMR systems preserve genome integrity through correcting mismatches and small insertion/deletion loops generated during DNA replication. Most hypermutant CRC have defects in MMR systems. The most important components of the MMR system are the MutS and MutL complexes (MSH2 and MSH6 form MutSα; MSH2 and MSH3 form MutSβ; MLH1 and PMS2 form MutLα; MLH1 and PMS1 form MutLβ; MLH1 and MLH3 form MutLγ). As MSH2 and MLH1 are proteins shared by MutS and MutL complexes, gene mutations will completely delay all MMR activities, resulting in the accumulation of insertion/deletion mutations in the repetitive nucleotide sequence regions of coding and non‐coding microsatellites.^16^, ^17^, ^18^, ^19^ Template and primer chains easily slip during replication, and this mismatch cannot be repaired in MMR defective cells. Thus, the number of duplicate units between the template and the newly synthesised chain is incorrect. MSI can lead to alternations in the translation reading framework and induce frameshift peptide (FSP). When an insertion/deletion mutation causes a frameshift in the C‐terminal amino acid sequence of a protein, the produced FSP has no protein functions due to the truncation of the protein. The newly emerging protein is antigenic to the patient's immune system, which is called a neoantigen.^20^ FSP uses major histocompatibility complex (MHC) class I and class II molecules as substrates for antigen presentation. When presented on the cell surface, the neoantigens can serve as the target of CD4^+^ helper T lymphocytes and CD8^+^ cytotoxic T lymphocytes (CTL) for tumour infiltration, thus enhancing the immunity to cancer and paving the way for immunotherapy targeting neoantigens^21^, ^22^ (Figure 2). Particularly, TMB has become an emerging clinical biomarker. TMB mainly refers to the number of somatic mutations in each coding region of the tumour genome, rather than tumours with a high frequency of synonymous somatic mutations. Tumours with high TMB tend to have more neoantigens, thus generating ‘new’ and highly immunogenic targets for the immune system.^23^, ^24^

### 1.3 The identification and validation of neoantigens

Neoantigens can trigger tumour‐specific immune responses and promote tumour eradication. Conversely, the failure to generate an appropriate immune response in the tumour microenvironment (TME) leads to cancer progression. The inactivated response is attributed to these two aspects. First, despite of hundreds of non‐synonymous point mutations in the tumour, sporadic mutations produce true neoantigens. It has been reported that only 0%–5% of mutant sequences in tumour can produce immunogenic neoantigen peptides.^25^, ^26^, ^27^, ^28^ The main explanation is that peptides derived from SNVs are similar to their unmutated counterparts, which are non‐immunogenic.^29^ Furthermore, dysregulated signalling pathways inhibit anti‐tumour immunity and promote cancer progression.^30^, ^31^ Therefore, the accurate prediction and prioritisation of neoantigen candidates is a fundamental and challenging task.^32^

**FIGURE 3:**
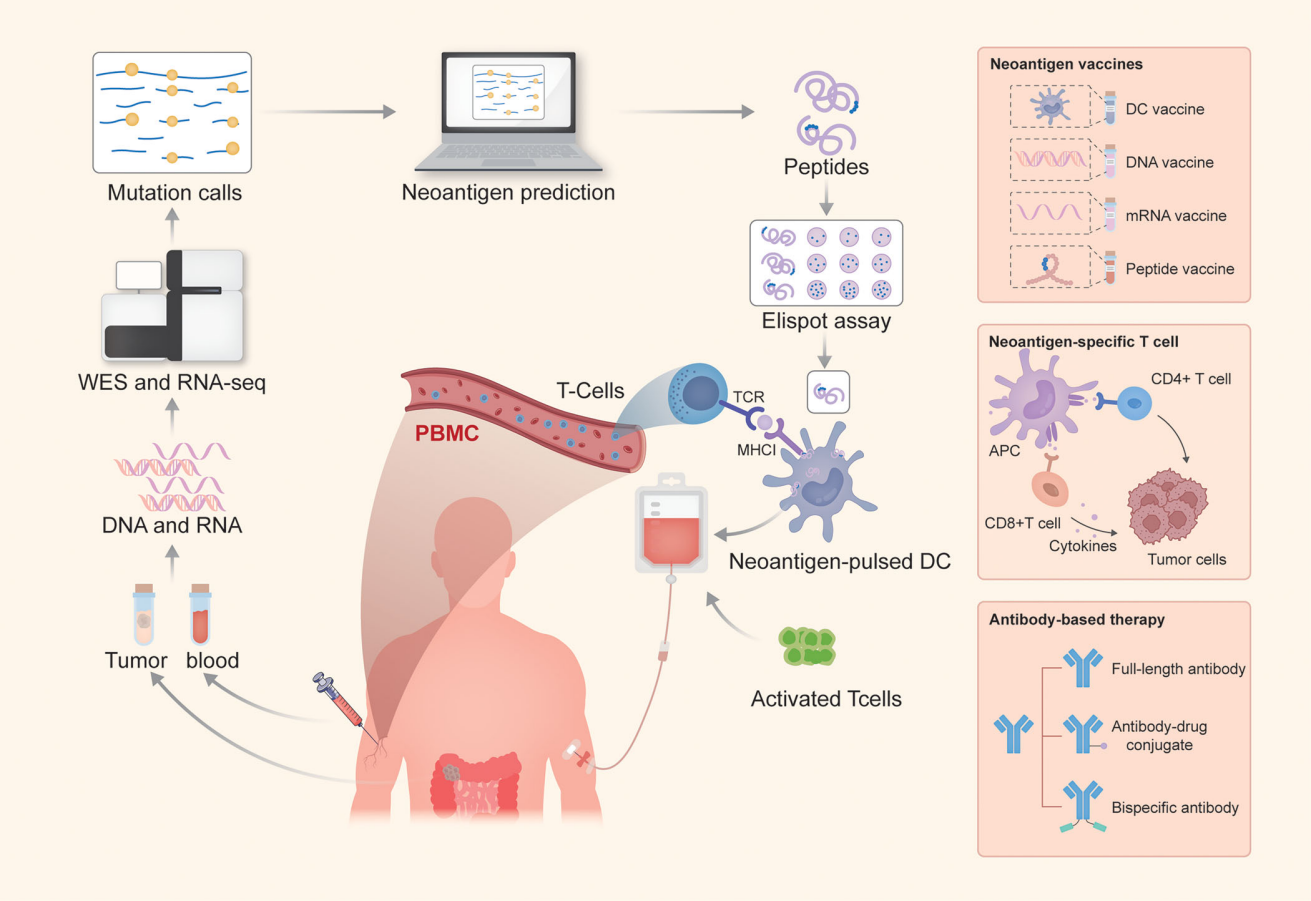
The workflow and classification of neoantigen‐based therapy for colorectal cancer (CRC). The process of individual therapeutics of neoantigen‐based therapy includes: (i) WES data and RNA‐seq of tumour and blood; (ii) mutant peptide calling using a set of somatic mutations and splicing variants; (iii) HLA binding prediction; (iv) T‐cell recognition prediction; and (v) neoantigens pulsed DC or activated neoantigen‐specific T cells are transfused into the body. Neoantigen‐based therapy includes neoantigen vaccine, neoantigen‐specific T‐cell and antibody‐based therapy.

The performances of potential candidate neoantigens depend on several key factors: (i) the mutation is expressed at the protein level; (ii) the mutant protein is processed into a suitable peptide for presentation by MHC molecules; (iii) binding affinity between mutant peptides and MHC molecules; and (iv) affinity between the new peptide/MHC complex and the T‐cell receptor (TCR) of responding T cells. The current workflow of neoantigen prediction can be divided into five steps. The first step is to sequence cancer cells using whole‐genome sequencing (WGS), whole‐exome sequencing (WES) or RNA‐seq to find out tumour‐specific somatic mutations, including SNV, indels, fusion and so forth. This is followed by variant invocation, human leukocyte antigen (HLA) typing and interaction predictions between HLA‐neoantigen‐TCR based on sequencing data. Finally, in vitro experiments are conducted to verify the predictions and screen for neoantigens with high quality^33^, ^34^ (Figure 3). Genomics‐based in silico prediction, mass spectrometry analysis and structure‐based prediction are three main methods to predict tumour‐specific neoantigens. Genomics‐based in silico prediction usually predicts neoantigens in two ways. One is to predict the binding affinity of the mutant peptide to HLA molecules, and the other is to predict the probability that the mutant peptide will be presented to the cell surface as a neoantigen. Presently, there are two kinds of algorithm models. Affinity prediction model combines the binding affinity data between peptides obtained in experiments and HLA. The model is constructed by neural network to predict the affinity between mutant peptides and corresponding HLA‐I and HLA‐II types and screen out peptides with high affinity. Based on the convolutional neural network (CNN) deep learning framework, Pe and Hsu's team has built a predictive model named iConMHC, which analysed the properties of the physical and chemical interactions (such as the contact potential and distance of folded proteins) between neoantigens and MHC to predict the binding affinity between peptide antigens and specific MHC genes.^35^ While the mass spectrum data prediction model is a deep learning model based on the mass spectrum sequencing data of the tumour cell surface and the HLA type information.^36^ Pyke et al. used 25 monoallelic cell lines to generate allele‐specific immunopeptidomics data and created a Systematic HLA Epitope Ranking Pan Algorithm (SHERPA) to predict MHC peptide binding and presentation.^37^ However, there are some drawbacks in the algorithm prediction. Peptides with high affinity do not necessarily be presented as neoantigens eventually, suggesting that the prediction of neoantigens has a high false‐positive rate. Meanwhile, the prediction accuracy of MHC class II epitopes is limited because most of the current prediction pipelines do not screen tumour antigen based on affinity prediction results of MHC class II molecules. The main reasons can be summarised into two aspects. First, there is a lack of binding affinity training data of MHC class II neoantigens. For example, the data of MHC class II ligands in the Immune Epitope Database and Analysis Resource (IEDB) database is much less than that of MHC class I ligands.^38^ Second, the peptide‐binding groove of MHC‐II is open at both ends, allowing the binding of long peptides of variable lengths. The peptide region on both sides of the core binding part of MHC‐II molecules can also affect the binding of peptides.^39^ These characteristics contribute to the complex identification of immunogenic MHC‐II epitopes and make it difficult to model MHC class II ligands. By utilising mass spectrometry (MS), antigenic peptides on the cell surface are eluted from HLA molecules, and the T‐cell response is assessed by evaluating cytokine production, activation labelling expression, and tetramer‐staining using candidate neoantigen peptides. This method greatly reduces the candidate range of neoantigens without bias.^40^, ^41^ Different from genomics‐based methods that only provide neoantigen prediction, mass spectrometry can reveal neoantigens derived from autologous cell mutants and those induced by peptide splicing mediated by proteasome.^42^, ^43^ Newey et al. established patient‐derived organoids (PDO) from patients with CRC as an in vitro model to investigate the neoantigen presentation using MS immunopeptidomics.^44^ However, the sensitivity of this method is low, and only the peak peptide can be detected, which makes some peptides with low expression levels easy to be ignored. Considering that multiple factors are involved in tumour neoantigen predictions, based on the existing prediction tools, bioinformatics pipelines are developed to simplify the process of neoantigen identification for different clinical purposes. For example, pVACtools can identify mutant peptides from different sources, integrate multiple data, prioritise predicted peptides and provide an end‐to‐end solution for the prediction of tumour neoantigens.^45^ TIminer integrated a set of bioinformatics tools to analyse RNA‐Seq data and somatic DNA mutations of a single sample. TIminer can process raw RNA sequencing data and extract information with de novo antigen prediction.^46^ Additionally, MuPeXI, TSNAD, Open Vax, pTuneos, ScanNeo and other neoantigen prediction channels have been also applied.^47^, ^48^, ^49^, ^50^, ^51^ Therefore, by combining genomics‐based prediction with high‐throughput HLA‐ligandome mass‐spectrometry analysis, the performance of the neoantigen discovery programme can be significantly improved to identify the required neoantigens. Structure‐based prediction can reveal the importance of peptide structure and physicochemical properties, as well as the importance of posttranslational modification, such as phosphorylation, citrullination and glycosylation. Besides, this method can generate predictions applicable to all types of MHC and TCR receptors, thus alleviating the limitations of small datasets with rare MHC alleles.^52^, ^53^ Lanzarotti et al. used a force‐field method to score a given TCR with p:MHC, and immunogenicity was estimated by calculating the interaction energy between TCR and each p:MHCII complex. Meanwhile, the predictive performance depends on the ability to accurately predict peptide MHC binding and model the structure of TCR:p:MHC complexes.^54^ Riley et al. developed a programme for accurate and rapid modelling of non‐peptide structures that bind to common class I MHC type HLA‐A2 and applied it to analyse datasets containing thousands of immunogenic, non‐immunogenic and non‐HLA‐A2‐binding peptides.^52^ Therefore, the combination of structure‐based methods and NGS data‐based MHC binding prediction can significantly improve the accuracy of immunogenic peptide selection, which is particularly important in the research of peptide‐based neoantigen targeted therapy.^55^

Many clinical studies have shown that some neoantigen vaccines predicted by computer failed to effectively exert anti‐tumour immunity. Therefore, the experimental verification of tumour neoantigen epitopes is required to screen out neoantigens that can exert better tumour immunity. The screening and verification of neoepitopes are often carried out by conventional immunoassay, including TCR profile‐based analysis and patient memory T‐cell analysis. interferon gamma (IFN‐γ) ELISPOT is the most used assay to verify the immunogenicity of various antigenic peptides. It predicts the response to tumour neoantigens by quantitative analysis of IFN‐γ released by activated T cells after the restimulation with the same antigens.^56^, ^57^ The study of TCR profiles currently focuses on the identification and characterisation of complementarity‐determining region 3 (CDR3) sequences of unique T‐cell clones. Current studies have shown the potential of TCR repertoire profiling as a biomarker of clinical response in patients with pancreatic cancer receiving immunotherapy, and there is a correlation between the size and diversity of the patient's TCR repertoire and the patient's response to immunotherapy.^58^ Memory T‐cell‐based neoantigen profiling also has the potential to accelerate the pace of personalised cancer therapy with the advantage of identifying tumour neoantigens that truly elicit an immune response against the tumour. Liu et al. detected tumour antigen‐specific T cells through interaction‐dependent fucose‐biotinisation and observed that TSA reactive CD8^+^ TIL exhibited a dysfunction phenotype with substantial proliferation and tumour‐specific killing ability.^59^ A study has shown that patients with cancer have immune response to 99% of antigen peptides after vaccination, while the candidate antigens obtained by computer prediction only have immune response to 60% of antigen peptides.^60^

However, the firm prediction of neoantigens is still a challenge. Recently, new prediction tools for neoantigens have been developed, but some effective prediction tools have not been incorporated into the formal analytical workflow. Several variants except SNVs and indels have been identified as sources of neoantigens, but there is little predictive support for them in the current prediction pipeline. Additionally, the low accuracy of class II HLA typing algorithm also prevents the extensive prediction of class II neoantigens. Meanwhile, the low proportion of immunologically significant neoantigens and the arduous, expensive and time‐consuming identification of these immunogenic neoantigens pose the dilemma in the prediction and screening of tumour neoantigens, which will also be a new direction for future exploration and improvement.^34^

## 2 THE NEOANTIGEN‐BASED THERAPY IN CRC

### 2.1 Effects of neoantigen‐induced T cells

T lymphocytes play an important role in the immune response of CRC. Frameshift mutations are associated with tumour‐infiltrating CD8^+^ T‐cell density, and neoantigen‐specific cytotoxic T cells can be observed in patients with these mutations.^61^ Frameshift mutations can produce mutant proteins to generate neoantigens that are recognised by the immune system as non‐self, and trigger an effective T‐cell response against cancer cells.^13^ It initially activated antigen‐presenting cells (APCs), especially dendritic cells (DCs), which recognise and capture antigens through phagocytosis and microphagocytosis. Simultaneously, when neoantigens are presented, MHCI/II and co‐stimulator molecules on the surface of DC upregulated due to the production of interleukin (IL)‐12 and chemokines. Neoantigens are transported to the endoplasmic reticulum by associated transporters (TAP) and bind to MHC‐I molecules on the cell surface to form the TCR‐pMHC complex (TCR‐peptide‐MHC complex) and present to the cell surface. The DCs loaded with neoantigens are recognised by the T‐cell receptor of CD8^+^ T cells to produce effector T cells and memory T cells to trigger T‐cell immune response. Tumour destruction is induced by the tumour toxicity and production of certain cytokines IFN‐γ (interferon‐γ) and TNF‐α (tumour necrosis factor‐α).^62^, ^63^, ^64^, ^65^ Of note, neoantigen‐specific CD4^+^ T cells may also play a role in anti‐tumour immunity. CD4^+^ Th cells may activate DC through CD40/CD40L interaction to enhance antigen cross‐presentation and produce many cytokines, including IFN‐γ and TNF‐α, thereby coordinating with CD8+CTL and promote the inflammatory environment. This is named the ‘help’ role in the generation of anti‐tumour cytotoxicity CD8^+^ T‐cell response (Figure 2). It has also been reported that neoantigen‐specific CD4^+^ T cells had cytotoxic gene expression after therapeutic vaccines and CD4^+^ T cells can direct kill tumour cells expressing MHC‐II.^40^, ^66^, ^67^, ^68^, ^69^ However, the specific mechanism of neoantigen‐specific CD4^+^ T cells induced by the vaccine still needs further study.^70^

### 2.2 Neoantigen targeted therapy in CRC

The treatment of CRC mainly focuses on surgical excision and postoperative comprehensive treatment, including radiotherapy, chemotherapy, targeted therapy and immunotherapy. With the development of cancer immunotherapy, neoantigens, a class of TSA, have been regarded as emerging immunotherapeutic targets and hold promise for a variety of solid cancers (Table 1). Importantly, neoantigens are being explored for anti‐tumour therapy in CRC (Table 2). Neoantigen‐based targeted therapy can be divided into neoantigen vaccine, neoantigen‐specific T‐cell and antibody‐based therapy (Figure 3).

**TABLE 1:** The completed clinical trials of personalised neoantigen‐based therapy in solid cancers.

Type of therapy	Tumour types	Study phase	Therapeutic strategies	Adjuvant therapy	Patient number	Clinical trail numbers
Peptide vaccine	NSCLC, CRC, pancreatic cancer, shared neoantigen‐positive solid tumours	Phase I/II	GRT‐C903 GRT‐R904	Nivolumab Ipilimumab	39	NCT03953235
Peptide vaccine	Pancreatic cancer	Phase I	iNeo‐Vac‐P01	GM‐CSF	7	NCT03645148 (71, 72, 73)
Peptide vaccine	NSCLC, MSS CRC, gastroesophageal adenocarcinoma, urothelial carcinoma	Phase I/II	GRT‐C901 GRT‐R902	Nivolumab Ipilimumab	29	NCT03639714 (74)
Peptide vaccine	Neoplasms	Phase I	Neopeptides	–	30	NCT04509167
Peptide vaccine	Melanoma	Phase I	Neopeptides	Poly‐ICLC	20	NCT01970358 (75)
Peptide vaccine	Bladder cancer	Phase I	PGV 001	Atezolizumab Poly‐ICLC	10	NCT03359239
Peptide vaccine	NSCLC	Phase I	NEO‐PV‐01	Pembrolizumab Carboplatin Pemetrexed	38	NCT03380871
Peptide vaccine	Bladder cancer, melanoma, skin cancer, NSCLC	Phase I	NEO‐PV‐01	Nivolumab	34	NCT02897765 (76, 77)
Peptide vaccine	Cutaneous melanoma, NSCLC, squamous cell carcinoma of the head and neck, urothelial carcinoma, renal cell carcinoma	Phase I/II	GEN‐009	Nivolumab Pembrolizumab	24	NCT03633110
Peptide vaccine	Solid tumour	Phase I	ASV® AGEN2017	QS‐21 Stimulon	3	NCT03673020
Peptide vaccine	Metastatic non‐squamous NSCLC	Phase I	NEO‐PV‐01	Pembrolizumab	38	NCT03380871
DNA Vaccine	Metastatic hormone‐sensitive prostate cancer	Phase I	ROSTVAC‐V ROSTVAC‐F	Nivolumab Ipilimumab	19	NCT03532217
DNA vaccine	Cutaneous melanoma	Phase I	IFx‐Hu2.0	–	7	NCT03655756
DC vaccine	Breast cancer	Phase I	Neoantigen pulsed DC	–	5	NCT04105582
DC vaccine	Breast cancer	Phase I	Peptide pulsed DC	–	9	NCT04879888 (78)
CTL transfer therapy	Solid cancer	Phase I	TSA‐CTL	Cyclophosphamide Fludarabine	11	NCT02959905 (79, 80)
Neoantigen‐specific T cell	Cutaneous melanoma	Phase II	Aldesleukin	Cyclophosphamide Fludarabine phosphate	11	NCT01807182

Abbreviations: CRC, colorectal cancer; DC, dendritic cell; MSS, microsatellite stability; NSCLC, non‐small‐cell lung cancer.

**TABLE 2:** The summarised clinical trials of neoantigen targeted therapy in CRC.

Trail number	Tumour	Intervention	Adjuvant therapy	Study phase	Patients	Estimated/actual completion date	Type of therapy	Status
NCT01885702	MSI‐positive CRC	Frameshift‐derived neoantigen‐loaded DC	/	Phase I/II	25	2022‐12	DC vaccine	Active, not recruiting
NCT04912765	HCC or CRLM	Neoantigen pulsed DC vaccine	Nivolumab	Phase II	60	2025‐05	DC vaccine	Recruiting
NCT05235607	Advanced melanoma, bladder cancer, CRC	Tumour antigen‐sensitised DC vaccine	/	Phase I	60	2023‐12	DC vaccine	Not yet recruiting
NCT04147078	Advanced gastric cancer, lung cancer, HCC, CRC	DC vaccine	/	Phase I	80	2023‐06	DC vaccine	Recruiting
NCT03639714	Metastatic NSCLC, MSS CRC, gastroesophageal adenocarcinoma, metastatic urothelial cancer	GRT‐C901 GRT‐R902	Nivolumabipilim‐umab	Phase I/II	214	2023‐03	Peptide vaccine	Completed
NCT05141721	Metastatic CRC	GRT‐C901/GRT‐R902	Atezolizumab	Phase II/III	665	2027‐03	Peptide vaccine	Recruiting
NCT03552718	Solid tumours including CRC	YE‐NEO‐001	/	Phase I	16	2025‐12	Peptide vaccine	Active, not recruiting
NCT03265080	Metastatic solid tumours including CRC	ADXS‐NEO	/	Phase I	5	2020‐11	Peptide vaccine	Terminated
NCT05243862	MSS CRC	PolyPEPI1018	Atezolizumab	Phase II	28	2026‐06	Peptide vaccine	Active, not recruiting
NCT05078866	Lynch syndrome	Nous‐209 vaccine	/	Phase I/II	45	2025‐07	Peptide vaccine	Recruiting
NCT05130060	Metastatic CRC	PolyPEPI1018	Trifluridine and tipiracil hydrochloride	Phase I	15	2023‐07	Peptide vaccine	Completed
NCT04117087	Metastatic MSS CRC	KRAS peptide vaccine	Nivolumab Ipilimumab	Phase I	30	2024‐12	Peptide vaccine	Recruiting
NCT04853017	Solid tumours including CRC	ELI‐002 2P	/	Phase I	18	2026‐03	Peptide vaccine	Active, not recruiting
NCT04799431	Pancreatic cancer metastatic CRC	Neoantigen vaccine	Poly‐ICLC retifanlimab	Phase I	12	2023‐05	Peptide vaccine	Withdrawn
NCT02600949	Pancreatic or CRC	Peptide vaccine therapy	Imiquimod, pembrolizumab sotigalimab	Phase I	150	2025‐05	Peptide vaccine	Recruiting
NCT03480152	Metastatic melanoma or epithelial cancer	mRNA‐based vaccine targeting neoantigens	/	Phase I/II	5	2019‐11	mRNA vaccine	Terminated
NCT03468244	Advanced esophageal squamous carcinoma, gastric adenocarcinoma, pancreatic adenocarcinoma, CRC	Personalised mRNA tumour vaccine	/	Phase I	24	2021‐12	mRNA vaccine	Unknown status
NCT03953235	Metastatic NSCLC, MSS CRC, pancreatic cancer, shared neoantigen‐positive tumours	GRT‐C903 GRT‐R904	Nivolumab Ipilimumab	Phase I/II	144	2023‐12	Shared neoantigen cancer vaccine	Completed
NCT02757391	Cholangiocarcinoma, CRC, esophageal carcinoma, gastric carcinoma, pancreatic adenocarcinoma	CD8^+^ T cells against personalised peptide antigens	Pembrolizumab	Phase I	1	2020‐10	Adoptive cell transfer therapy	Terminated
NCT03431311	CRC	Adoptive cell therapy	/	Phase I/II	1	2019‐06	Adoptive cell transfer therapy	Terminated
NCT05576077	Solid tumours including CRC	TBio‐4101	Pembrolizumab	Phase I	30	2025‐06	TIL transfer therapy	Recruiting
NCT05292859	Solid tumours including CRC	Neoantigen‐specific TCR‐T cells	/	Phase I/II	180	2039‐04	Adoptive cell transfer therapy	Recruiting
NCT05194735	Solid tumours including CRC	Neoantigen‐specific TCR‐T cells	Aldesleukin (IL‐2)	Phase I/II	180	2029‐03	Adoptive cell transfer therapy	Active, not recruiting
NCT03970382	Solid tumours including CRC	NeoTCR‐P1 adoptive cell therapy	/	Phase I	21	2022‐08	Adoptive cell transfer therapy	Suspended
NCT03190941	Metastatic or unresectable RAS G12V‐expressing cancer including CRC	Anti‐KRAS G12 V mTCR	Cyclophosphamide, Fludfarabine aldesleukin	Phase I/II	110	2028‐06	Adoptive cell transfer therapy	Recruiting
NCT03526835	Metastatic CRC	MCLA‐158	/	Phase I/II	360	2024‐06	Antibody‐based therapy	Recruiting
NCT03035253	Metastatic CRC	OMP‐305B83	FOLFIRI FOLFOX	Phase I	16	2018‐12	Antibody‐based therapy	Terminated

Abbreviations: ACT, adoptive cell therapy; CRC, colorectal cancer; CRLM, colorectal liver metastasis; DC, dendritic cell; HCC, hepatocellular carcinoma; MSI, microsatellite instability; MSS, microsatellite stability; TCR, T‐cell receptor.

#### 2.2.1 Neoantigen vaccine

Neoantigen‐based vaccines can be divided into peptide vaccines, nucleic acid vaccines and DC vaccines with different technical routes and clinical characteristics.^33^ Cancer vaccines can induce a strong immune response to one or more specific antigens, enhancing local T‐cell infiltrations and exerting cytotoxic effects on cancer cells expressing these antigens (Figure 3).

Peptide vaccines, the most common form of the vaccine, consist of recombinant or purified proteins. The peptide vaccine can activate pre‐existing neoantigen‐specific T cells and induce many specific T lymphocytes.^15^, ^81^ For example, short peptide vaccines usually bind directly to MHC‐I molecules to generate corresponding T‐cell responses (Figure 2). However, most cells expressing MHC‐I molecules are not specifically used for antigen presentation, which leads to difficulty in T‐cell activation by short peptide vaccines after administration.^82^, ^83^, ^84^ Polypeptide vaccines usually requires uptake and processing by specialised antigen‐presenting cells before delivery. Peptide vaccines not only avoid the development of CTL tolerance, but also provide potential MHC‐II class epitopes involved in CD4^+^ T‐cell responses.^85^, ^86^, ^87^ In a phase I clinical trial, the safety and immunogenicity of FSP neoantigens caused by AIM2, HT001 and TAF1B mutations were evaluated in 22 dMMR patients with CRC. The results showed that one frameshift peptide vaccine induced both humoral and cellular immune responses without serious vaccine‐related adverse reactions in all patients.^88^ However, the application of peptide vaccines is limited due to their unique peptide epitopes, low molecular weight, simple degradation and short half‐life.^89^, ^90^

**FIGURE 4:**
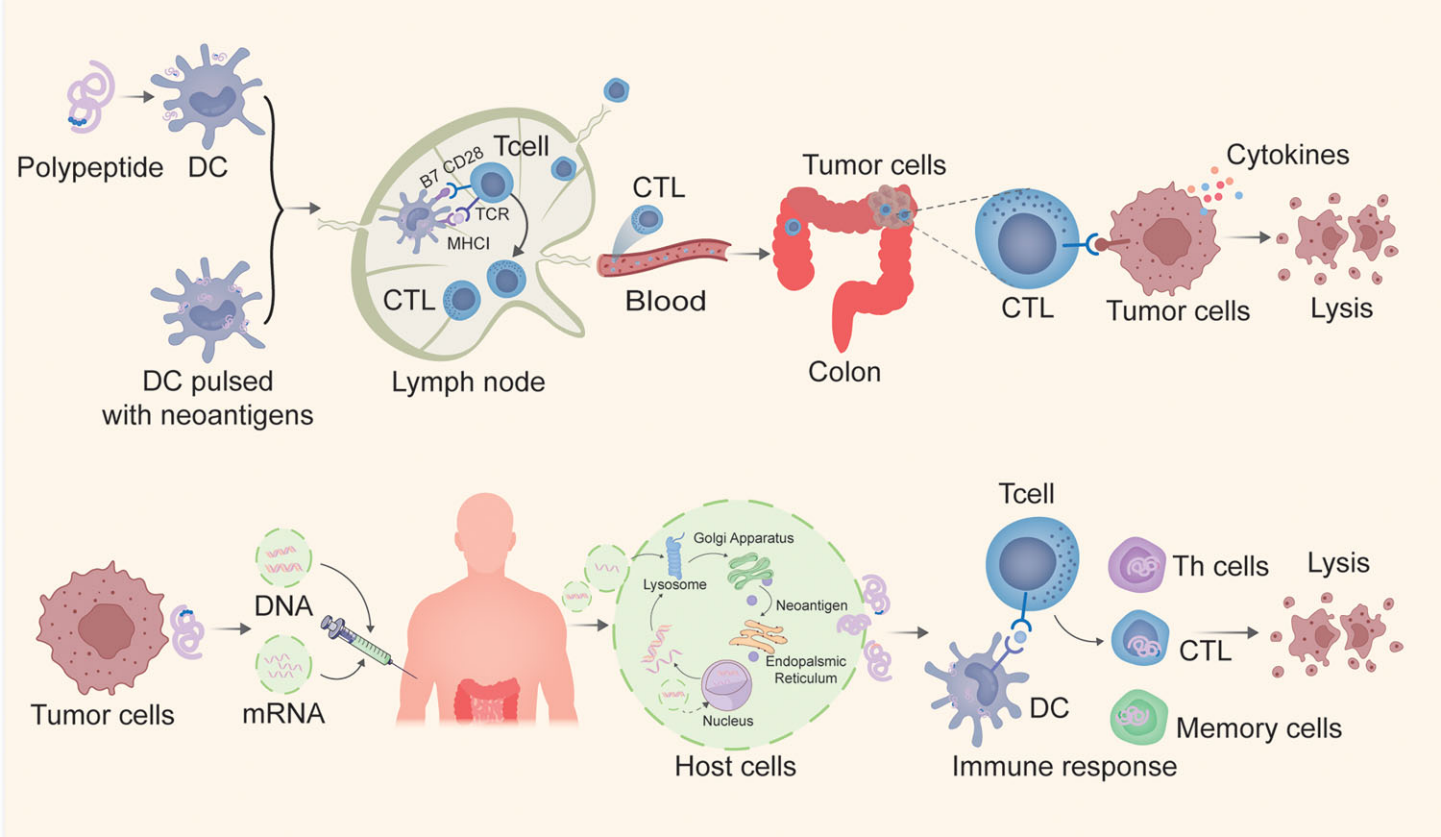
The mechanism of peptide vaccine, DC pulsed with neoantigens and nucleic acid vaccines in colorectal cancer (CRC). Neoantigen‐based vaccines can be divided into peptide, nucleic acid and DC vaccines with different technical routes and clinical characteristics. The peptide vaccine can activate pre‐existing neoantigen‐specific T cells and induce many specific T lymphocytes. DNA vaccines can spontaneously induce T‐cell response against tumour and inhibit tumour growth. mRNA does not need to enter the nucleus and alter the host cell's genome, but can be directly translated into antigens by ribosomes, thereby triggering targeted anti‐tumour cell immune responses. DC vaccine promotes the presentation of neoantigens and amplify neoantigen‐specific T cells. DC, dendritic cell; CTL, cytotoxic lymphocyte.

Nucleic acid vaccines are mainly divided into DNA vaccine and mRNA vaccine.^91^ Nucleic acid vaccines offer greater advantages over peptide vaccines in terms of efficacy, reduced design and manufacturing time, and manufacturing scalability and reliability. DNA vaccines are a common form of neoantigen vaccines due to their simple manufacturing and low cost. DNA vaccines can spontaneously induce T‐cell response against tumour and inhibit tumour growth. Veisi Malekshahi et al. found that the use of plasmid DNA vaccine carrying carcinoembryonic antigen (CEA) can stimulate tumour‐bearing mice to produce CEA‐specific T cells and antibody responses, and the level of anti‐CEA‐specific IgG antibody in the serum of tumour‐bearing mice also increases. Furthermore, the secretion of IFN‐γ, IL‐2 and lymphocyte proliferation also stimulate T‐helper‐1, thus exerting anti‐tumour immune effect.^92^ mRNA vaccines have been shown to safely induce neoantigen‐specific T‐cell responses and stimulate the anti‐tumour immune response.^93^, ^94^, ^95^ Compared to other types of vaccines, mRNA vaccines not only avoid insertion mutations and abnormal transcription, but also reduce the risk of side effects caused by the conversion of DNA into protein and the reduction of biodegradable components of DNA.^95^, ^96^ For example, after the synthetic messenger RNA is delivered to the cytosol of body cells, it does not need to enter the nucleus and change the genome of host cells. RNA is immediately translated into antigens by ribosomes, thus causing targeted anti‐tumour cell immune response (Figure 4). Notably, the mRNA vaccine has the advantages of low synthesis cost, short synthesis cycle, encoding multiple antigen sequences simultaneously and lack of MHC haplotype restriction. Furthermore, due to the inherent immunogenicity of mRNA, it can induce an optimal immune response with a low dose, making messenger RNA vaccine an effective and promising vaccine platform.^97^ Using three different tumour mouse models, Kreiter et al.^98^ found that there were significant proportion of non‐synonymous mutations in immune‐related genes and most of these immune‐related gene mutation products can be recognised by CD4^+^ T cells. Then, the researchers established a process for screening tumour‐specific mutations by exon sequencing. According to the expression levels and binding abilities to MHC‐II, they used bioinformatic methods to prioritise the selected tumour‐specific mutations and quickly synthesise multi‐epitope mRNA vaccines for cancer immune‐targeted therapy. Sahin et al.^99^ demonstrated that mRNA vaccines containing neoantigens in combination with ICIs can induce CD4^+^ T‐cell and CD8^+^ T‐cell responses. A series of studies have proved that the multi‐epitope mRNA vaccines can effectively inhibit tumour growth with a good therapeutic effect.^98^ However, because RNA enzymes are abundant in blood and body tissues, mRNA is easily degraded during delivery before entering cells. Therefore, efficient delivery of RNA to tumour cells is critical for the effectiveness of RNA vaccines.

DC vaccine is an effective immunotherapy option because of its simple preparation principle, low toxicity, lack of invasive procedures and potential long‐term anti‐cancer effects. DC vaccine promotes the presentation of neoantigens and amplify neoantigen‐specific T cells^100^ (Figure 4). The neoantigen‐pulsed DC vaccine has considerable anti‐tumour activities in patients with advanced relapse.^101^, ^102^ The new neoantigens presented by HLA‐A*02:01 broadens the antigenic breadth and clonal diversity of anti‐tumour immunity in patients with advanced melanoma.^100^ More and more researchers have paid attention to the development and application of neoantigen vaccines. A new era of neoantigen immunity is coming.

#### 2.2.2 Neoantigen‐specific T cells

Adoptive cell transfer therapy is a type of immunotherapy in which immune cells, such as TILs, chimeric antigen receptor T cells (CAR‐T) and T‐cell receptor (TCR)‐modified T cells, are transferred to patients. T cells that specifically recognise neoantigens and induce anti‐tumour responses are ideal vectors for the adoptive cell therapy (ACT). The application of neoantigens in ACT is a promising strategy. Yu et al.^103^ found that neoantigen‐containing peptides (SEC11A‐R11L and ULK1‐S248L) were identified from HLA‐A0201^+^PW11 via neoantigen‐reactive T‐cell (NRT) cytotoxicity assay in vitro and in vivo experiments in tumour‐bearing mouse models. Compare with the corresponding natural peptides, SEC11A‐R11L and ULK1‐S248L are more effective in initiating antigen‐specific CTL responses. Meanwhile, adoptive transfer of NRTs inoculated with these two mutant peptides can effectively inhibit tumour growth. Parkhurst et al.^104^ treated three patients with metastatic CRC with transgenic autologous T lymphocytes expressing a murine TCR against human CEA. Significant reductions in serum CEA levels were observed in all patients, and lung and liver metastases of one patient were reduced. Another clinical trial also proposed that autologous transfer of HLA‐C*08:02‐restricted KRAS G12D‐reactive polyclonal CD8^+^ T‐cell populations successfully cured patients with metastatic CRC.^105^ These studies indicate the promise of neoantigen‐specific T cells in CRC therapy.^106^

#### 2.2.3 Antibody‐based therapy

Neoantigen‐based antibody therapies have been used in tumour therapy. TCR‐mimic (TCRm) antibodies or mutation‐associated neoantigens (MANA)‐specific antibodies are usually converted into full‐length antibodies, antibody–drug conjugations (ADC) and bispecific antibodies (BsAbs) in various therapeutic forms. These antibodies focus on the pMHC complex to recognise intracellular neoantigens and drive the specific activities of neoantigens to exert anti‐tumour effects^107^, ^108^ (Figure 3). Hsiue et al.^109^ developed a BsAb based on a p53 mutation that binds the p53^R175H^ peptide/HLA‐A*02:01 complex and the TCR‐CD3 complex to T cells with high affinity, which overcomes the lack of neoantigen presentation and selectively redirected T cells to recognise cancer cells with mutant peptides. Low's team developed TCRm antibodies targeting the p53_125‐134_/HLA‐A24:02 complex through an ADC therapeutic form and coupled the cytotoxic drug (PNU‐159682) to inhibit the growth of colon cancer cells expressing mutant p53 in vitro and in animal models.^110^ The affinity of TCRm antibody to polypeptide‐HLA molecules is much better than that of natural TCR. To prevent interference from unrelated HLA components, phage, yeast and genetic platform are used to screen appropriate TCRm antibodies to achieve accurate and effective therapeutic effects.^111^, ^112^, ^113^ Therefore, the development of antibody‐targeted therapies against public neoantigens has great prospects.^108^

### 2.3 The prognostic role of neoantigens in CRC

Neoantigens play a pivotal role in predicting the therapeutic efficacy and forecasting the prognosis of tumour with patients. Both the number and immunogenicity of neoantigens impact the therapeutic responses and prognosis of patients with cancer.^114^ In patients with advanced non‐small cell lung cancer (NSCLC) after treatment with PD‐1 inhibitor (pembrolizumab), the exon sequencing results showed that patients with higher tumour neoantigens and less intratumoural heterogeneity had higher PD‐L1 expression, sensitivity to immune checkpoint suppression therapy and longer overall survival time.^115^ Anagnostou et al.^114^ found that patients with NSCLC who were resistant to anti‐PD‐1/anti‐CTLA‐4 antibodies lost some of their neoantigens and produced new mutations. These lost neoantigens had stronger MHC binding than the remaining neoantigens. Tumours with increased burden of mutation‐associated neoantigens had significant therapeutic responses to ICIs. Similarly, approximately 15% of CRC are identified as MSI‐CRC with abundant mutation‐derived neoantigens that trigger a robust anti‐tumour response. Accordingly, ICIs are highly effective in patients with MSI‐CRC with remarkable and durable tumour responses.^116^ In contrast, the remaining 85% of CRC are microsatellite stable (MSS) and mainly exhibit the CIN phenotype.^117^ Patients with MSS CRC do not respond to PD‐1‐based immunotherapy.^118^ The low TMB and lack of immune cells infiltration are main reasons for the insensitivity of CRC patients to immunotherapy.^119^ Notably, nearly 3% of CRC is classified as MSS with high TMB. These MSS patients with high TMB may respond favourably to ICI‐based therapy.^120^ The underlying mechanism for the generation of high TMB in MSS CRC is still not clear. Neoantigen‐derived epitopes (neoepitopes) on HLA‐I have also been observed in MSS CRC.^44^ It suggests complex tumour intrinsic or microenvironmental may be responsible for the poor immunogenicity of MSS CRC, and further mechanistic identification is needed.^121^, ^122^ Therefore, to achieve better anti‐tumour response via neoantigen‐based therapy, it is important to balance the quantity and quality of tumour neoantigens. High‐quality antigenic peptides are prioritised by selecting peptides with better attributes based on the results of machine learning approaches. Typically, the top 20 personal mutated peptides with high‐affinity binding of autologous HLA molecules per patient were selected to synthesise the vaccine or reagents.^72^, ^75^, ^123^

### 2.4 The combination therapy of neoantigen‐based therapy with ICIs and traditional therapy in CRC

Neoantigen‐based therapy mediate tumour clearance mainly by inducing cytotoxic T cells. However, tumour cells have multiple immune escape mechanisms. Besides, the tumour microenvironment also interferes with the functions of immune cells and even suppresses the immune response, which limits the anti‐tumour effect of neoantigen‐based therapy in patients with cancer.^124^, ^125^, ^126^, ^127^, ^128^, ^129^, ^130^, ^131^ To enhance the efficacy of neoantigen‐based therapy in CRC, a series of researches are exploring the potential of combination neoantigen‐driven therapies with other traditional immunotherapy, chemoradiotherapy, antiangiogenic agents and oncolytic viruses (OVs). The mechanism is mainly to improve the immune microenvironment in the tumour. The combination of neoantigen‐based therapy with ICIs is mechanistically complementary. Neoantigen‐based therapy stimulates the immune system by activating CD8^+^ T cells and CD4^+^ Th1 cells. IFN‐γ produced by the T cells subsequently increases the expression of PD‐L1 on tumour cells and inhibits the function of the neoantigen vaccine. After the immune system was activated, the expression of CTLA‐4 on the surface of T cells also increased. Then, CTLA‐4 binds to the immune system ligand B7‐1/B7‐2 and exerts an immunosuppressive effect on antigen‐presenting cells. Checkpoint inhibition therapy involves the use of specific monoclonal antibodies, including anti‐CTLA‐4, anti‐PD‐1 and anti‐PD‐L1 antibodies. These antibodies bind to the immune checkpoint protein of T cells to eliminate inhibition of T‐cell functions by tumour cells.^132^, ^133^, ^134^, ^135^ Importantly, neoantigen‐based immunotherapy is complementary to ICIs as it has no specific requirement for patient's MSI status nor TMB.^12^, ^136^ Several clinical trials of combination of ICIs and neoantigen‐based therapy are being conducted in patients with CRC (Table 2).

Traditional therapies, such as radiation and chemotherapy, can also enhance the effect of neoantigen vaccines. Chemotherapy or radiotherapy can induce the release of multiple antigens from tumour cells. When the amount of neoantigens in a tumour is too low to activate the T‐cell response, a combination of neoantigen vaccines and chemotherapy can disturb this dilemma (Figure 2). Radiotherapy can also enhance the infiltration of T cells in tumour tissue, thus stimulating the specific anti‐tumour immune responses. Besides, chemoradiotherapy can also reduce the immunosuppression in the tumour microenvironment and enhance the therapeutic effect. These combined strategies suggest that immune‐mediated and radiation‐driven systemic therapy models will also be a new prospect in the field of personalised therapy.^137^, ^138^

OVs can directly lyse tumour cells, resulting in the immunogenic cell death and the release of soluble antigens, which promotes anti‐cancer immunity under tolerable safety conditions (Figure 2). OVs can induce the release of tumour neoantigens and epitope spreading to promote the priming of neoantigen‐specific CD8^+^ T cells by BATF3^+^ DCs, thus inhibiting distant, uninfected tumours.^139^, ^140^, ^141^ Despite there is no direct evidence of the combination of neoantigen‐based therapy in the treatment of CRC, the ability of OVs to increase neoantigen presentation and expand the neoantigen‐specific T‐cell repertoire is being used in the preclinical research to enhance the anti‐cancer effects of neoantigen‐based therapy. A novel adeno‐associated viral (meAAV) neoantigen vaccine modified with TLR9 inhibitory fragments, PD‐1 traps and PD‐L1 mirnas was designed to prolong the persistence of meAAV. The modified vaccine can induce strong antigen presentation and maintain the neoantigen‐specific CTL response in CRC. Meanwhile, these functional PD‐1 traps and PD‐L1 mirnas also overcome the host PD‐1/PD‐L1 inhibitory mechanism, thereby improving the therapeutic effect of radiotherapy and leading to the complete elimination of colorectal tumour in mice.^142^

To maximise therapeutic efficacy of neoantigen‐based therapy in the CRC, the combination with other therapeutic strategies becomes a hot topic. Mechanically, the integration of immunotherapies with diverse mechanisms of action allows for the simultaneous targeting of all stages of the cancer immune cycle, including antigen release and presentation, initiation and activation of immune cells, transportation and infiltration of immune cells into the tumour, as well as recognition and eradication of cancer cells. Additionally, the administration of chemotherapy, radiation, targeted therapy and oncolytic viral therapies induces cell death, thereby augmenting the generation and release of neoantigens, and further amplifying the anti‐tumour immune response (Figure 2). Furthermore, ICIs facilitate the infiltration of immune cells into tumours, leading to the elimination of malignant cells.^108^ Overall, the main principle of the combination strategies is to enhance or restore tumour‐specific T cell‐mediated anti‐tumour immunity. The efficacy and safety of the combination of patient‐specific cancer vaccines and ICIs was evaluated in the first‐line metastatic setting of metastatic MSS CRC. An individualised, heterologous chimpanzee adenovirus (ChAd68) and self‐amplifying mRNA (samRNA)‐based neoantigen vaccine in combination with nivolumab and ipilimumab improved the overall outcome of several patients with metastatic MSS CRC (NCT03639714).^74^ Then, larger randomised studies were proposed to further explore the efficacy and safety of individual neoantigen vaccines in MSS CRC. In an ongoing randomised phase 2/3 study, estimated 700 patients with MSS CRC will receive a total of six administrations of GRT‐C901/GRT‐R902 (patient‐specific vaccines) plus ipilimumab co‐administered only with the first dose of GRT‐C901 and GRT‐R902. All patients will receive atezolizumab in addition to maintenance therapy of fluoropyrimidine and bevacizumab according to standard of care (NCT05141721). Several clinical trials were ongoing to assess the efficacy and safety of the combination of neoantigen‐based immunotherapy and ICIs. Published results from these trials are highly awaited.

## 3 THE CHALLENGE OF NEOANTIGEN‐BASED THERAPY IN CRC

CRC is a heterogeneous disease with a variety of clinical and biological characteristics that lead to differences in disease progression and treatment response.^143^ Therefore, for patients with CRC, tumour heterogeneity has become a major challenge in diagnosis and treatment. It is mainly reflected in inter‐patient heterogeneity, inter‐tumour heterogeneity and intra‐tumoural heterogeneity.^144^ As a result of tumour heterogeneity, various clinicopathological parameters, including TNM pathological staging, histological staging and specific molecular markers, such as RAS and BRAF mutation status, have been proposed to evaluate prognosis and determine personalised treatment options for individual patients. Neoantigens, which exhibit high specificity for each patient, can be selectively targeted by cancer vaccines to elicit de novo T‐cell responses, thereby enabling the attainment of personalised precision treatment.^145^ With a personalised approach to cancer treatment, patients can benefit from effective treatment with minimal side effects.^146^, ^147^ However, there are still limited data on its application in patients with CRC. Clinical trials with large sample size are expected to verify that neoantigens can effectively eliminate tumour cells in patients with CRC.

Tumour antigen selection is the key to success of any cancer vaccine.^148^ For an ideal tumour antigen, a big size and a high affinity for binding MHC molecules are essential to ensure the adequate immune cell recognition and killing. Neoantigens with strong binding affinity to MHC molecules can be predicted by tumour exon sequencing.^115^, ^149^ Despite the detection of tens of thousands of mutations in tumour patients, the prediction of neoantigens is limited to only two digits. It should be noted that not all peptides can be synthesised and commercially produced in vitro when considering the production of predicted peptides. Furthermore, only a small number of valuable neoantigens can elicit an immune response in clinical settings.^29^ Additionally, the length and amino acid number of antigens must be considered to optimise the breadth of CD8^+^ and CD4^+^ T‐cell responses. Typically, CD8^+^ T cells recognise 8–11 amino acid peptides in MHC‐I, while CD4^+^ T cells recognise 12–15 amino acid peptides bound to MHC‐II.^150^, ^151^ Therefore, ensuring the appropriate length and quantity of neoantigens is another major challenge in the realisation of tumour immunotherapy.

The emergence of tumour immune escape and immunosuppression also makes the neoantigen‐based treatment difficult. First, tumour cells are derived from the body's own cells. Only a small number of abnormally expressed proteins have immunogenicity. The immunogenicity of spontaneous tumours is usually weak, and it is difficult to induce effective anti‐tumour immune response because of the escape from the recognition and killing of the immune system. Second, tumour cells with the loss or low expression of MHC‐I class molecules have defects in antigen presentation and affect the activation of tumour antigen‐specific CD8^+^ T cells. Meanwhile, killer cell immunoglobulin‐like receptors (KIRs) on the surface of NK cells can recognise MHC‐I molecules (such as HLA‐G, HLA‐E) that are abnormally expressed on the surface of tumour cells, thus activating inhibitory signals to inhibit the cytotoxic effects of NK cells. Furthermore, tumour cells can secrete IL‐10, TGF‐β and other cytokines, which inhibit the function of immune cells and facilitate tumour cells to escape from immune attack. Some tumour cells can also express FasL and combine with activated Tc‐expressing Fas to mediate tumour antigen‐specific T‐cell apoptosis. Moreover, tumour cells highly express multiple oncogene products, such as the Bcl‐2 family, which can resist tumour cell apoptosis mediated by activated Tc to facilitate the abnormal proliferation of tumour cells.

The neoantigen‐based therapy also needs to consider the factor of immune interference. Oliveira's group proposed that HLA class II^neg^ tumours can indirectly activate tumour‐specific immunosuppressive T regulatory (T_Reg_) cells, thus causing immunosuppression.^152^ The similarity in TCR specificity between neoantigens and TEx TCR leads to a notable phenotype alteration in cytotoxic CD4^+^ T cells among melanoma patients with abnormal HLA class II expression. This alteration results in a substantial depletion of these cells, consequently eliciting putative immunosuppressive neoantigen‐specific T_Reg_ clones. The emergence of immune interference may consequently counteract the expected high immunogenicity of extremely mutated tumours.^152^, ^153^ Therefore, in future neoantigen screening of CRC, we need to pay attention to the existence of immune interference, and select neoantigen vaccines that can exert a positive anti‐tumour effect, which is a new research direction of neoantigen immunotherapy.^154^

The sources of tumour antigens mainly include TAA and TSA.^155^, ^156^ TAA, such as CEA, is not an optimal target for immunotherapy. First, because TAA is also expressed in some normal tissues, immunotherapy against TAA may activate the immune response in non‐target tissues and cause severe autoimmune toxicity, such as severe hepatitis, colitis, rapid respiratory failure, renal insufficiency and even death. Neoantigens are expressed only by tumour cells and trigger tumour‐specific T‐cell responses, thereby preventing ‘off‐target’ damage to non‐tumour tissue.^157^ Second, the efficacy of TAA‐based therapeutic vaccines is hindered by the presence of central and peripheral immune tolerance mechanisms. This is compounded by the fact that the high‐affinity TCR for TAA is preferentially consumed through positive selection in the thymus, resulting in a lower affinity of TCR for TAA than neoantigens. Neoantigens are specific epitopes derived from somatic mutations. Neoantigens can evade T‐cell central tolerance to their epitopes, thereby facilitating the enhancement of tumour‐specific immune responses.^70^, ^108^, ^158^ It is worth noting that the administration of a neoantigen vaccine can stimulate the production of antigen‐specific memory T cells and offer a long‐term prevention from the tumour recurrence.

Current targeted therapies for neoantigens include personalised neoantigens for each patient or common neoantigens expressed in many patient cancers.^159^ As most tumour mutations are not shared among patients, the personalised neoantigens were ‘tailored’ for each patient's tumour tissue mutation to ensure that each patient has its specific vaccine.^160^, ^161^ Recently, public neoantigens have become a compelling therapeutic target.^107^ Compared with personalised neoantigens, treatment methods based on public consume less resources and time, bypassing many of the limitations of personalised neoantigens, including the complex production process inherent in personalised immunotherapies, while avoiding prolonged production time that increases the likelihood of disease progression before treatment is initiated.^107^, ^108^, ^162^ Public neoantigen vaccines derived from KRAS mutations have been put into clinical use. Vaccine strategies against TP53 and BCR/ABL fusion public tumour antigens have been implemented and have successfully generated new antigen‐reactive T cells. The clinical effects of vaccine against TP53 and BCR/ABL fusion public tumour antigens are being researched.^107^, ^163^ Of note, adoptive transfer of autologous TIL that is responsive to KRAS p.Gly12Asp neoantigen can result in the tumour regression in patients with metastatic CRC.^164^ Progress in identifying more public neoantigens is critical to extend the application of these approaches to more patients. However, no patient is likely to have more than one or a few public neoantigen treatment targets. Instead, cancers often have multiple private neoantigens. Therefore, the combination of public neoantigens and personalised antigens may enhance precise immunotherapies against the most common oncogene alternations in CRC.

The process of pre‐screening to confirm the efficacy of neoantigens incurs substantial costs, time constraints and financial burdens in their production. Numerous clinical studies investigating tumour neoantigens lack adequate time to pre‐screen the immunological characteristics of the anticipated candidate tumour neoantigens prior to treatment. Consequently, extensive animal models and well‐controlled clinical trials are necessary to address these concerns.^165^ The development of individual therapeutics centred on neoantigens entails a highly tailored and personalised approach, encompassing gene sequencing, design, validation and ultimate production. The entire treatment is time consuming and expensive.^100^, ^166^, ^167^, ^168^ Therefore, solving these problems is the key to popularising neoantigens widely in the treatment of CRC.

## 4 PERSPECTIVE AND PROSPECT

The special anti‐tumour effect and low incidence of adverse events indicated that the immunotherapy targeting neoantigens is a considerable therapeutic strategy for CRC. Neoantigen‐based therapy alone or combined with other treatment strategies is used in many clinical trials and is entering a new era (Table 1). Many problems remain to be addressed and optimised for prospective therapy based on neoantigens in CRC. The rapid pace of tumour‐specific mutations makes immunological targets elusive. Comprehensive identification strategies are needed to address the trade‐off between precision drug therapy and large‐scale production of personalised neoantigen therapies. Additionally, the combination of biopsy images and NGS sequencing (including TCR analysis) is required to overcome the tumour heterogeneity of neoantigen therapy barriers. Furthermore, the tumour immune‐suppressive microenvironment should be considered to achieve robust T‐cell responses. Altogether, the integration of immunobiology, medicine, informatics and artificial intelligence holds the potential to enhance the efficacy of tumour therapy through the utilisation of neoantigens.

Ongoing investigations are currently examining the potential of neoantigens in the management of CRC. The utilisation of viral carriers and the integration of nanotechnology in vaccine development have introduced a novel avenue for antigen therapy. Consequently, the treatment approach for CRC is progressively transitioning towards precision medicine and combination therapy. By studying neoantigens in CRC, we can enhance our comprehension of the underlying mechanisms of CRC pathogenesis, identify novel therapeutic targets and facilitate the development of personalised treatment approaches, thereby paving the way for precision medicine in CRC.

## CONFLICT OF INTEREST STATEMENT

The authors declare they have no conflicts of interest.
